# Deficiency of Daily Calcium and Vitamin D in Primary School Children in Lviv, Ukraine

**DOI:** 10.3390/ijerph19095429

**Published:** 2022-04-29

**Authors:** Andrii Tytusa, Jutyna Wyszyńska, Marta Yatsula, Serhiy Nyankovskyy, Artur Mazur, Katarzyna Dereń

**Affiliations:** 1 Department of Pediatrics #1, Danylo Halytsky Lviv National Medical University, 79010 Lviv, Ukraine; a.tytusa@gmail.com (A.T.); dr.yatsula@gmail.com (M.Y.); nianksl@gmail.com (S.N.); 2 Institute of Health Sciences, Medical College of Rzeszow University, 35-959 Rzeszow, Poland; kderen@ur.edu.pl; 3 Institute of Medical Sciences, Medical College of Rzeszow University, 35-959 Rzeszow, Poland; drmazur@poczta.onet.pl

**Keywords:** calcium, children, deficiency, dietary intervention, vitamin D

## Abstract

This study was conducted to determine the prevalence of calcium and vitamin D deficiency in school children aged 6 to 11 years in Lviv (Ukraine). The aim of this study was also to assess the effect of nutritional intervention and supplementation on serum calcium and vitamin D levels in children diagnosed with calcium deficiency. A 3 day diet was analyzed in 172 children. In 56 children with a deficiency of calcium and/or vitamin D, the level of total calcium, ionized calcium, and 25-hydroxyvitamin D in blood, as well as the calcium content in hair samples, was determined. Thirty children with confirmed calcium and/or vitamin D deficiency underwent a dietary intervention to increase calcium and vitamin D intake for 3 months. The study group (*n* = 15) was provided with a nutritional correction by modifying their diet with supplementation of calcium and vitamin D. The control group (*n* = 15) received only a diet modification. Decreased daily calcium intake was observed in 50.68% of boys and 70.71% of girls. A decreased level of daily vitamin D consumption was found in 84.93% of boys and 96.97% of girls. Among 56 children with insufficient daily consumption of calcium and/or vitamin D, 26.8% had reduced levels of total calcium in the blood, 16.1% had reduced levels of ionized calcium in the blood, and 48.2% had reduced levels of vitamin D. After 3 months of correction, normal calcium level was found in the hair of 10 students (66.7%) from the study group and in three subjects (20.0%) from the control group. Modification of the diet along with supplementation of calcium and vitamin D seems to be a more effective method for increasing the level of calcium among children aged 6 to 11 years.

## 1. Introduction

Nutrition is one of the most important factors that ensure the maintenance of health, physical efficiency, longevity, creativity, and disease prevention [1,2]. Consumption of insufficient amounts of nutrients affects not only the physical development of children, but also their cognitive and psychosocial development [3]. According to studies conducted in different countries, a significant number of children at different ages have latent or severe deficiencies of various nutrients including proteins, fats, vitamins, and minerals [4,5].

Calcium is one of the key minerals needed to ensure many physiological processes in the body of the child, including regulation of hormone secretion, participation in muscle contraction and nerve impulse transmission, immune system function, mental performance, and bone development [6,7,8]. Ensuring proper calcium intake can prevent the development of various disorders, help minimize growth problems, prevent osteoporosis and osteopenia, and protect against future bone fractures [9,10]. Calcium metabolism is closely dependent on vitamin D, parathormone, and calcitonin levels. Vitamin D stimulates intestinal absorption of calcium and phosphorus, as well as regulates serum calcium levels, maintaining the proper mineralization of the skeleton [11]. Insufficient intake of calcium or vitamin D in children can lead to disorders of bone mineralization, as well as disorders of the muscular, nervous, immune, and other systems [12].

The pediatric population has an increased need for vitamin D and calcium due to the rapid growth and increase in bone mass during this period [13]. Studies in various regions of the world have shown that the apparently healthy pediatric population is deficient in calcium and vitamin D, and that the incidence of rickets and osteomalacia is increasing [13,14,15]. The high prevalence of vitamin D deficiency among school-age children warrants intervention for proper nutritional support [16]. Furthermore, the percentage of individuals meeting the recommended dietary allowance (RDA) decreases as children reach adolescence [17].

The possibilities of effective correction of dysmicroelementosis by modification of nutrition or by use of micronutrient supplementation remain insufficiently studied [18]. According to Kvashnina, prevention and correction of hypocalcemia in healthy children of primary school age should be carried out using a balanced diet including calcium and vitamin D supplementation [19].

A high rate of vitamin D deficiency in the pediatric population has a multifactorial origin, the most important of which is considered insufficient intake related to dietary habits [20]. Moreover, the efficacy and practicability of a dietary intervention aiming to normalize calcium and vitamin D intake is still largely unknown [21]. As such, there is a need for effective interventions that ensure that children meet the RDA of vitamin D and calcium.

There are limited studies on vitamin D and calcium supplementation in Ukrainian children, more so regarding an adequate dose of vitamin D supplementation in prepubertal children. Therefore, the aim of this study was to evaluate the prevalence of calcium and vitamin D deficiency in school children aged 6 to 11 years in Lviv (Ukraine). The aim of the study was also to assess the effect of nutritional intervention and supplementation on serum calcium and vitamin D levels in children diagnosed with calcium deficiency.

## 2. Materials and Methods

### 2.1. Participants

This randomized interventional study was conducted during the 2019–2020 academic year among primary schoolchildren in public elementary schools 34 and 10 in Lviv, Ukraine.

The inclusion criteria consisted of children (1) who were 7 to 11 years old, (2) attending primary education at one of the two selected schools, and (3) whose parents or caregivers provided written parental consent and child assent prior to data collection. The exclusion criteria were (1) a clinical diagnosis of chronic disease, (2) absence from school on the day of the examination, or (3) an incomplete nutritional record.

**Figure 1:**
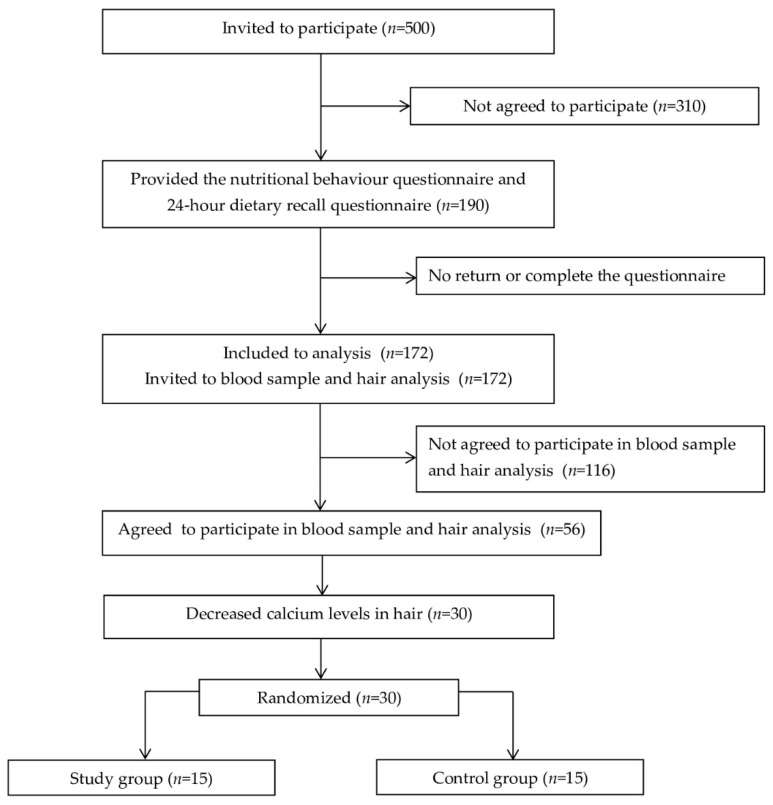
Flow diagram of participant recruitment.

The study involved 190 students: 81 boys (42.6%) and 109 girls (57.4%). According to age, children were divided as follows: 6–7 years of age—41 children (21.6%), 8 years of age—38 children (20%), 9 years of age—36 children (19%), 10 years of age—51 children (26,8%), and 11 years of age—24 children (12.6%). A flow diagram of participant recruitment is presented in Figure 1.

### 2.2. Measurements

#### 2.2.1. Nutritional Behavior

To assess the nutritional behavior of the participants, a questionnaire was used which was self-reported by the children together with their parents. The questionnaire contained questions on basic eating habits and eating behavior of the child at home and at school. The questionnaires were distributed at parent meetings in the respective schools after coordination with the school administration and the Department of Education of Lviv. Moreover, a 24 h dietary recall was used to assess individual characteristics of the diet and daily nutrient intake of participants [22]. The dietary recall was collected over 3 days (two weekdays and one weekend day). Parents carefully wrote down all the food items that the child ate in a diary, noting their weight and volume. Parents were given special electronic scales for weighing food. The results were processed using a licensed program Dietplan 7 (UK). Calcium and vitamin D were calculated and included in the analysis. The results were interpreted according to the recommended daily allowance (increased, decreased, or normal intake) for each macro- or micronutrient. The norms for the parameters analyzed were as follows: (a) calcium: 800 mg; (b) vitamin D: 5 ug; (c) total calcium in blood: 2.2–2.7 mmol/L; (d) ionized calcium in blood: 1.16–1.32 mmol/L; (e) vitamin D in blood: >20 ng/mL; (f) calcium in hair: 300–700 mg/kg. Values of the analyzed parameters above the norm were marked as ‘increased’, while those below the norm were marked as ‘decreased’. This program allowed determining the daily intake of all essential nutrients, taking into account the age, sex, and reference values of nutrient intake and caloric content of foods recommended by the Committee of Medical Aspects of Food Policy (1991) and adapted to norms and standards of Ukraine (norms of physiological needs of the population of Ukraine in basic nutrients and energy, Order of the Ministry of Health of Ukraine No. 1073 from 03.09.2017). A total of 172 correctly completed diaries were received (response rate of 90.5%).

#### 2.2.2. Determination of the Level of Calcium and Vitamin D

As the study was conducted in the autumn and winter, the analysis did not take into account the sun exposure and BMI. Children, who were found to be deficient or subnormal in calcium and/or vitamin D intake according to the diet analysis, were referred for additional evaluation of total ionized calcium and 25-hydroxyvitamin D levels in the blood, as well as calcium content in hair. A total of 56 parents agreed to this evaluation.

Total calcium in venous blood was assessed by colorimetric analysis using an analyzer and a Cobas 6000 test system (Roche Diagnostics, Rotkreuz, Switzerland). To determine ionized calcium in venous blood, an ion-selective analysis using an analyzer and test system–AVL 9180 (Roche Diagnostics, Switzerland) was used. Determination of 25-hydroxyvitamin D (25- (OH) D) levels in venous blood was performed by the means of an enzyme-linked immunosorbent assay using an analyzer and test system—EUROIMMUN (Lubeck, Germany). Biochemical studies were performed in the network of Synevo laboratories.

Hair calcium was determined using an atomic absorption spectrophotometer C-115.M1 (certificate of calibration No. UA/37/261118/001543 from 22 November 2018). Approximately 1.5 g of hair was collected by trimming the hair at the nape of the neck at a distance of approximately 5 cm near the scalp. The hair length ranged from 1 to 3 cm. To minimize contamination transfer, clipping of the hair samples was performed using plastic disposable forceps, fresh stainless-steel scissors, and disposable vinyl examination gloves [23]. Hair samples were prepared and analyzed by an atomic absorption spectrophotometer according to the method of MB 77-02010793:2019 “Measurement of the mass concentration of calcium in the hair by atomic absorption spectrophotometry” [24]. The analysis was performed in duplicate. Metrological characteristics of the method were as follows: working range—from 50.0 mg/kg to 1000.0 mg/kg; quantitative limit (LOQ)—50.0 mg/kg; relative extended measurement uncertainty (U)—21.0%; recurrence (r)—8.0%; calibration characteristic stability index (K)—12.0%. The study of calcium content in hair was conducted in the Central Research Laboratory of Danylo Halytsky Lviv National Medical University. Decreased calcium levels in the hair were found in 30 children, which we interpreted as a confirmatory factor for calcium deficiency in the child’s body.

### 2.3. Intervention

Subsequently, 30 children with low hair calcium (less than 300 mg/kg) were randomized into two groups of 15 children. The study group had an average of 181.55 ± 86.95 mg/kg of hair calcium, and the control group had an average of 164.43 ± 80.88 mg/kg. The age of the participants in the study group and control group was similar. The children from the study group were provided nutritional correction by modifying their diet with increased consumption of products containing calcium and vitamin D, calcium supplements at a dose of 500.0 mg, and vitamin D at a dose of 5.0 µg (200.0 IU) once a day for 3 months. For children in the control group, nutritional correction was performed only by a 3 month diet modification without added supplementation.

In order to evaluate the effectiveness of corrective measures in children of both groups, a repeated study of the calcium content in hair samples was performed after 3 months.

To confirm the adequacy of the sample size to the results obtained, an a posteriori analysis of strength for calcium in the hair for the experimental group was conducted. According to the results of calculation, the difference between the average levels of calcium in the hair before and after treatment in the experimental group yielded a Cohen’s *d* value of 1.28, interpreted as a “very large” effect size. At an α level of 0.05 and a sample size of 15 participants, the achieved 1 − β power was 99%.

### 2.4. Statistical Analysis

Interpretation of the obtained results was performed by methods of descriptive statistics with an estimate of the average value of indicators (mean), the value of the standard deviation (SD), the confidence interval (CI), and the criterion of the probability of error-free prediction (*p*). The analysis took into account relative and absolute values and the normality of the distribution. Given the correct distribution of variables to compare the two groups, a parametric method with Student’s *t*-test for independent samples was used. Under the condition of nonparametric distribution of variables, Fisher’s angular transformation method was used to compare the two groups. To determine the relationship between the indicators, Pearson’s correlation analysis (R) was performed. Statistical analysis was performed using the statistical application package STATISTICA For Windows v.6.1 (StatSoft, Tulsa, OK, USA), MS Excel Online (Microsoft, Redmond, WA, USA), and the Internet portal “Medical Statistics” http://www.medstatistic.ru/calculators.html (accessed on 10 September 2021). The level of statistical significance was set at *p* < 0.05.

### 2.5. Ethics

This study on children was ethically approved, and research was conducted taking into account the basic principles of the Helsinki Declaration on Biomedical Research and the provisions of GCH ICH, in accordance with bioethical standards (excerpt from protocol No. 1 of meeting of the commission on bioethics of Danylo Halytsky LNMU from 31 January 2018).

## 3. Results

The analysis of the nutritional value of daily diet established that the average value of daily calcium intake was 750.00 mg per day (603.00; 949.00) in boys and 646.00 mg per day (502.50; 744.50) in girls and depended on gender and age. Decreased levels of calcium intake depending on gender were observed in 37 (50.68%) boys and 70 (70.71%) girls (*p* < 0.01). In the age group of 6–7 years of age, calcium intake was reduced in 29 schoolchildren (76.32%); the following reductions were recorded in the remaining groups: 8 years of age—in 26 schoolchildren (70.27%), 9 years of age—in 11 (35.48%) schoolchildren, 10 years of age—in 27 schoolchildren (61.36%), and 11 years of age—in 14 students (63.64%) (*p* < 0.01; Table 1).

The average daily intake of vitamin D in schoolchildren was 2.59 µg (1.68; 4.08) in boys and 1.97 µg (1.19; 3.08) in girls and depended on gender. Decreased levels of vitamin D intake by sex were observed in 62 (84.93%) boys and 96 (96.97%) girls (*p* = 0.01) (Table 2). Among 56 children tested for calcium and vitamin D, a decreased level of calcium and vitamin D in blood serum was found in two (7.69%) boys and 13 (43.33%) girls (*p* < 0.01). Decreased serum ionized calcium levels were found in one (3.85%) boy and in eight (26.67%) girls (*p* < 0.01). Decreased serum vitamin D levels were observed in eight (30.77%) boys and 19 (63.33%) girls (*p* = 0.02). According to the obtained results, reduced calcium content in hair was observed in eight (30.80%) boys and 22 (73.30%) girls (*p* < 0.01).

**Figure 2:**
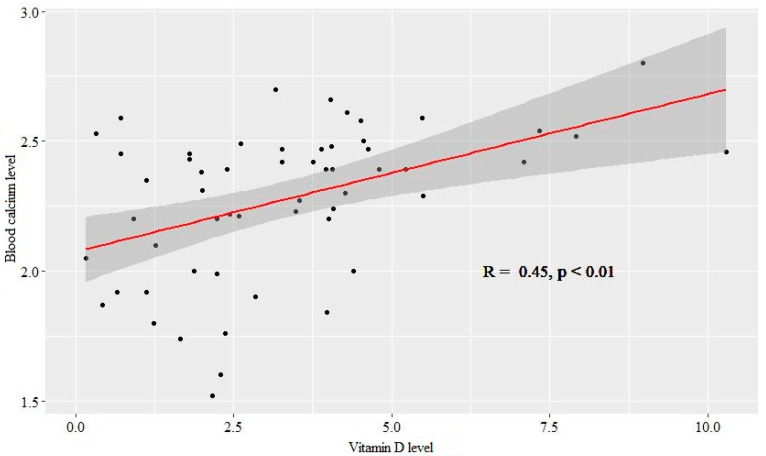
Correlation between the level of vitamin D intake and the level of calcium in the blood.

When comparing the dependence of calcium in the blood and nutrients consumed, high blood calcium was most often observed at high values of vitamin D (*R* = 0.45, *p* < 0.01) (Figure 2).

**Figure 3:**
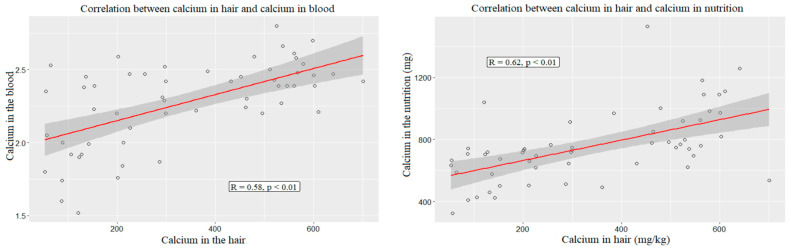
Correlation between calcium in hair and calcium in blood and nutrition.

The absolute level of calcium in hair correlated with the indicators of calcium in blood and in nutrition. There was a positive, strong, reliable correlation with blood calcium (*R* = 0.58, *p* < 0.01) and a positive, strong, reliable correlation with the amount of daily calcium intake (*R* = 0.62, *p* < 0.01 (Figure 3)).

After 3 months of follow-up, hair calcium content in children from both groups was remeasured. The average calcium content in hair from the control group was 185.14 ± 82.38 mg/kg (*p* > 0.05). In the study group, where, in addition to diet modification, children received calcium and vitamin D supplements for 3 months, a significant increase was noted in the average level of calcium in hair from 181.55 ± 86.95 to 293.60 ± 80.66 mg/kg (*p* < 0.01). Normal calcium level was found in the hair of 10 students (66.7%) from the study group, but only three subjects (20.0%) from the control group (Table 3).

## 4. Discussion

Evidence has shown a clear relationship linking nutrition, cognitive development, and academic achievements [25,26]. Data from recent studies indicated a fairly frequent inadequacy and imbalance of childhood nutrition both at home and at preschool educational institutions and schools. Deviations are expressed as insufficient protein content in the diet with excessive consumption of carbohydrates and fats, as well as deficiency of vitamins and microelements.

The results of our study showed the presence of significant deficiencies in the nutrition of students in two primary schools in city of Lviv. Lviv is a fairly large, modern Ukrainian city, with a population approaching one million and a developed school infrastructure. Disorders of eating behavior (irregular diet, decreased appetite, and insufficient consumption of dairy products, fish, vegetables, and meat) could cause a deficiency in vitamins and minerals, including calcium and vitamin D. Given the possibility of developing a deficiency in the consumption of certain nutrients in any diet, such children should be under the supervision of a nutritionist or an informed pediatrician to ensure adequate intake of all food ingredients with the use of supplements if necessary.

Calcium is an essential mineral that is involved in many physiological processes in the child’s body. Adequate amounts of lactose, vitamin D and C, fats, and proteins increase the bioavailability of calcium, and its bioavailability is reduced when consumed with products that contain oxalic and phytic acids [27,28]. Recommendations for calcium intake vary depending on age, gender, and some genetic and environmental factors. Adequate calcium intake is about 1000 mg/day for children aged 6–8 and about 1300 mg/day for children aged 9–18 [29,30].

Vitamin D plays a decisive role in the regulation of calcium absorption and excretion. Insufficient intake of calcium or vitamin D in children can lead to their deficiency, disorders of bone mineralization, and disorders of the muscular, nervous, immune, and other systems [31]. The optimal level of 25(OH)D in blood is associated with the prevention of diseases related to the immune system (asthma, type 1 diabetes), infectious diseases (respiratory infections, influenza), and cardiometabolic markers [32]. In order to prevent hypovitaminosis D, the Institute of Medicine (IOM) established an RDA of 600 IU/day for children 1–18 years old [33]. After reviewing the literature, the IOM concluded that levels of 25(OH)D 20 ng/mL meet the requirements of at least 97.5% of healthy individuals [33,34,35].

Low calcium in nutrition may not manifest itself clinically for a long time, but it often causes insufficient bone mass at an older age and in critical periods of life (first year, 1–2 years, 5–7 years, 12–14 years), leading to osteoporosis. Students’ daily calcium intake of up to 370 mg is accompanied by gradual growth retardation and weight loss. In children who received calcium in the amount of up to 230 mg per day, there was a significant decrease in bone density [36]. According to Kvashnina, prevention and correction of hypocalcemia in healthy children of primary school age should be carried out using a balanced diet and calcium and vitamin D supplements [19]. The American Academy of Pediatrics (AAP) determines that the main areas of overcoming calcium deficiency are (1) prescription of foods rich in calcium, (2) prescription of calcium supplements, and (3) use of vitamin D [37,38].

An analysis of the nutritional value of daily diets using the Dietplan 7 program found that the average daily calcium intake was 750.00 mg per day in boys and 646.00 mg per day in girls. According to the updated recommendations in Ukraine, the daily calcium intake of 6 year old students should be 600 mg/day, with the following recommendations for other age groups: 7–10 years old—1000 mg/day and 11–13 years old—1200 mg per day. Thus, we identified a significant deficiency of daily calcium intake by schoolchildren with food, which can lead to disruption of calcium accumulation. The obtained values of daily calcium intake were lower than both IOM and national recommendations [29]. Similar results were obtained in a study performed in Morocco, although they did not obtain a difference in results depending on gender [30]. According to data on Spanish schoolchildren, 25.8% of all schoolchildren studied presented with inadequate calcium intake. The prevalence of inadequate calcium intake was significantly higher for girls (*p* = 0.006) [39]. The calcium deficiency was higher in children from developing countries, affecting 79% of children aged 10 to 16 years in Bangladesh and 100% in Senegal [40].

We revealed a low level of calcium intake depending on gender. It was twice as common in girls, which was, to some extent, due to the peculiarities of their diet. In our study, we found a significant reduction in daily intake of vitamin D. Its average value was 2.59 µg (1.68; 4.08) in boys and 1.97 µg (1.19; 3.08) in girls. Vitamin D deficiency depended on gender and was observed in 96.97% of girls and 84.93% of boys (*p* = 0.01). Combined daily deficiencies of calcium and vitamin D were identified in a significant proportion of schoolchildren. Among Italian children at the average age of 10 years, a low vitamin D intake was observed in all subjects (median 0.79 µg/day), and it was associated with calcium intake lower than 70% of the dietary reference intakes in 55% subjects. After 4 months of nutritional intervention, a normalization of serum 25(OH)D levels was observed in all children in Group 1 (receiving dietary counseling to optimize daily calcium and vitamin D intake plus administration of calcium and vitamin D supplementation product) and in only one subject from Group 2 (receiving dietary counseling alone) [20].

Given the significant percentage of children with reduced daily calcium and/or vitamin D intake, the vitamin D and the total and ionized calcium levels were determined in 56 children whose parents consented to the study. It turned out that these indicators also depended on gender. Thus, the reduced level of total calcium in the blood was determined in 7.69% of boys and 43.33% of girls (*p* < 0.01), while the reduced level of ionized calcium in the serum was determined in 3.85% of boys and 26.67% of girls (*p* < 0.01). Serum vitamin D levels were also gender-dependent. Decreased serum vitamin D levels were observed in 30.77% of boys and 63.33% of girls (*p* = 0.02) in this group. Zhang et al. showed a blood calcium deficiency of 29.3% in Chinese children aged 1–10 years. In addition, they showed that seasons have a significant relationship with blood calcium levels, especially in girls. Children in winter, compared to children in spring and summer, had significantly lower calcium concentrations [41].

The level of trace elements in a child’s hair can be a fairly clear indicator of the body’s supply of these nutrients. Therefore, we performed the determination in the hair of children in this group. Decreased hair calcium (less than the reference values for this age: 300–600 mg/kg) was found in 30.8% of boys and 73.3% of girls (*p* < 0.01). The calcium level in hair correlated with blood calcium and daily calcium intake (*p* < 0.01). Studies have shown that the accumulation of elements in the scalp hair and blood of children varies according to age, sex, place of residence, ethnic and geographical origin, and nutritional status. A study by Ozmen et al. showed that measured calcium levels in whole blood or hair were lower compared to reference values [42].

The effectiveness of the use of dietary modification for 3 months only in schoolchildren with a certain deficiency of calcium in terms of calcium levels in the hair was unsatisfactory. Normalization of this indicator was observed in only 20% of children in this group. The use of a similar dietary correction with the simultaneous use of calcium and vitamin D supplementation allowed to normalize the calcium content in the hair of 66.7% of children. A systematic review of 14 studies on improving dairy food and calcium intake in children aged 5–12 years indicated that 10 were considered to be effective. Studies focusing on encouraging intake of dairy foods or calcium alone were all effective, compared with 55% of studies promoting dairy within the context of a healthy diet [43].

The question of how long to continue a modified diet and intake of calcium and vitamin D remains uncertain. According to researchers from China, calcium supplementation in children who had habitually low calcium intakes of 280 mg/day resulted in an increase in appendicular bone mass [44] similar to that reported from supplementation studies of children on much higher calcium intakes. As has been reported from a number of other calcium supplementation studies, the bone mass advantage was lost within 18 months of stopping the supplement [45].

Given the significant changes that have taken place in the organization of school nutrition in Ukraine in 2021, it is advisable to conduct new studies to assess the provision of schoolchildren with essential nutrients, both in large cities and in rural areas of different regions of Ukraine.

### The Limitations

The nutritional behavior questionnaire used in the study should be considered as a limitation of the study as it has not been validated. Therefore, results related to the use of this questionnaire should be treated with caution. Moreover, the results may be limited to some extent because all schools were located in one metropolitan area; in addition, the study sample was relatively small and not an ideal representation of the population of Ukrainian children. In addition, there were 11% students included in the study with special or different diets. Not excluding them from the study should be considered another limitation of the study.

## 5. Conclusions

In study participants, the deficiency of daily intake of calcium and vitamin D depended on gender and was more common in girls. In a fairly large proportion of children with insufficient daily intake of calcium and/or vitamin D, there was a low level of total and ionized calcium in the serum, as well as reduced calcium levels in hair. Modification of the diet along with supplementation of calcium and vitamin D seems to be a more effective method for increasing the level of calcium among children aged 6 to 11 years.

## Figures and Tables

**Table 1:** Frequency of reduced, normal, and increased levels of daily calcium intake, relative to the recommended daily dose in children.

Nutrient	Level of Consumption	Age (Years)					*p*
		7 *n* (%; CI)	8 *n* (%; CI)	9 *n* (%; CI)	10 *n* (%; CI)	11 *n* (%; CI)	
Calcium	Reduced	29 (76.32%; 62.80–89.83)	26 (70.27%; 55.54–85.00)	11 (35.48%; 18.64–52.33)	27 (61.36%; 46.98–75.75)	14 (63.64%; 43.54–83.74)	**<0.01**
	Normal	4 (10.53%; 0.77–20.28)	5 (13.51%; 2.50–24.53)	13 (41.94%; 24.56–59.31)	7 (15.91%; 5.10–26.72)	0 (0.00%; 0.00–0.00)	
	Increased	5 (13.16%; 2.41–23.91)	6 (16.22%; 4.34–28.09)	7 (22.58%; 7.86–37.30)	10 (22.73%; 10.34–35.11)	8 (36.36%; 16.26–56.46)	

CI—confidence interval; significant associations are highlighted in bold.

**Table 2:** Calcium and vitamin D levels in the analyzed samples.

Indicator	Level	Boys *n* (%; CI)	Girls *n* (%; CI)	*p*
Vitamin D intake	Reduced	62 (84.93%; 76.73–93.14)	96 (96.97%; 93.59–100.00)	**0.01**
	Normal	6 (8.22%; 1.92–14.52)	1 (1.01%; 0.00–2.98)	
	Increased	5 (6.85%; 1.05–12.64)	2 (2.02%; 0.00–4.79)	
Total blood calcium	Reduced	2 (7.69%; 0.00–17.93)	13 (43.33%; 25.60–61.07)	**<0.01**
	Normal	23 (88.46%; 76.18–100.00)	17 (56.67%; 38.93–74.40)	
	Increased	1 (3.85%; 0.00–11.24)	0 (0.00%; 0.00–0.00)	
Ionized blood calcium	Reduced	1 (3.85%; 0.00–11.24)	8 (26.67%; 10.84–42.49)	**<0.01**
	Normal	14 (53.85%; 34.68–73.01)	19 (63.33%; 46.09–80.58)	
	Increased	11 (42.31%; 23.32–61.30)	3 (10.00%; 0.00–20.74)	
Vitamin D in blood	Reduced	8 (30.77%; 13.03–48.51)	19 (63.33%; 46.09–80.58)	**0.02**
	Normal	18 (69.23%; 51.49–86.97)	11 (36.67%; 19.42–53.91)	
Calcium in hair	Reduced	8 (30.80%; 13.03–48.51)	22 (73.30%; 57.51–89.16)	**0.01**
	Normal	17 (65.40%; 47.09–83.67)	8 (26.70%; 10.84–42.50)	
	Increased	1 (3.80%; 0–11.23)	0 (0.00%)	

CI—confidence interval; significant associations are highlighted in bold.

**Table 3:** Indicators of calcium in the hair of children in the study groups at the beginning of observation and after 3 months of treatment.

Group	At the Start	After 3 Months	Difference	*p*
	Mean ± SD	Mean ± SD		
Control	164.43 ± 80.88	185.14 ± 82.38	20.71	0.07
Study	181.55 ± 86.95	293.60 ± 80.66	112.05	**<0.01**
Difference	17.12	108.46		
*p*	0.58	**<0.01**		

Significant associations are highlighted in bold.

## Data Availability

The data analyzed during this study are available from the corresponding author.
